# Substantiation of propitious “Enzybiotic” from two novel bacteriophages isolated from a wastewater treatment plant in Qatar

**DOI:** 10.1038/s41598-022-13171-8

**Published:** 2022-05-31

**Authors:** Ramya Ramadoss, Moza Al-Shukri, Basem Shomar, Valentin Alekseevich Ilyin, Annette Shoba Vincent

**Affiliations:** 1 Biological Sciences, Carnegie Mellon University Qatar, PO box 24866, Doha, Qatar; 2 Environmental Science Center (ESC), Qatar University, PO box 2713, Doha, Qatar; 3 Computational Biology, Carnegie Mellon University Qatar, PO box 24866, Doha, Qatar

**Keywords:** Bacteriophages, Protein sequence analyses, Bioinformatics, Protein purification, Environmental biotechnology

## Abstract

Lysin of bacteriophages isolated from a particular ecosystem could be inducted as a bio-controlling tool against the inhabiting pathogenic bacterial strains. Our study aims at both experimental and computational characterization of the identical lysin gene product inherent in the genomes of two novel *Myoviridae* bacteriophages, *Escherichia* Phage C600M2 (GenBank accession number OK040807, Protein ID: UCJ01465) and *Escherichia* Phage CL1 (GenBank Genome accession number OK040806.1, Protein ID: UCJ01321) isolated from wastewater collected from the main water treatment plant in Qatar. The lysin protein, evinced to be a globular *N*-acetyl-muramidase with intrinsic “cd00737: endolysin_autolysin” domain, was further expressed and purified to be experimentally validated by turbidimetric assay for its utility as an anti-bacterial agent. Comprehensive computational analysis revealed that the scrutinized lysin protein shared 85–98% sequence identity with 61 bacteriophages, all native to wastewater allied environments. Despite varied Host Recognition Components encoded in their genomes, the similitude of lysins, suggests its apparent significance in host–pathogen interactions endemic to wastewater environment. The present study substantiates the identical lysin from *Escherichia* Phage C600M2 and *Escherichia* Phage CL1 as propitious “enzybiotic”, a hybrid term to describe enzymes analogous to anti-biotics to combat antibiotic-resistant bacteria by in silico analysis and subsequent experimental validation.

## Introduction

The issue of water scarcity around the world is prevalent today and its sustainability and security are especially critical in middle eastern countries such as Qatar. With a population of more than 2.68 million as of December 2021, Qatar’s water consumption is one of the highest in the world per capita at 500 L per day^1^. Water available for use originates from sources such as abstraction of fresh and saline groundwater, seawater desalination and re-use of treated sewage effluent, with seawater desalination accounting for 99% of Qatar’s drinking water supply. As a result, Qatar has several water sustainability programs carried out by water authority: Kahramaa, among its top priorities. These programs are majorly focused on finding out major and minor chemical and microbial contaminants in drinking water. Wastewater treatment plants have been set up by the authorities to explore alternatives to desalination of seawater and abstraction of Qatar’s limited fresh groundwater resources. As of 2015, 34% of treated wastewater was used for agriculture irrigation and 16% for green space irrigation.

Despite of the continuous efforts, assuring adequate water quality still remains challenging^2^. Several recent research publications suggest that wastewater treatment plants worldwide confirm prevalence of bacterial communities with antibiotic resistance, irrespective of operational efficiency^3–6^. On the bright side, the same environment facilitates rapid discovery of bacteriophages predacious towards the native bacteria^7^ and could be enlisted as potent as bio-control^8^ for sustainable wastewater treatment. For instance, two novel *E. coli* bacteriophages isolated from the Zayandehrood River in Iran were used in wastewater treatment processes^9^.

These bacteriophages isolated from wastewater could also serve as a reservoir of lytic enzymes with the capability of anti-biotics, coined as “enzybiotics”^10^ to control drug-resistant pathogenic bacteria^11^. The lytic enzymes target pathogenic bacteria while leaving commensal microflora unaffected^12^ and have proven to be effective in various clinical applications as alternatives to replace antibiotics^13^. Moreover, lysins are highly stable and could be produced in large scale^14^ and do not spread Anti-biotic resistance genes through horizontal transfer, thus making them more reliable than bacteriophages for biocontrol and pharmaceutical applications^13^. For instance, endolysins isolated from phages predacious to contaminating drug-resistant pathogens have been proved as efficient “enzybiotic” agents for water treatment in aquacultures^15,16^.

Lysins induce rapid lysis of the bacterial peptidoglycan cell wall layer and initiate or culminate the infection cycle by either being part of the phage-tail, enacting localized degradation termed as the “lysis from without” phenomenon^17^, for phage-DNA entry or as endolysins, facilitating bacterial cell wall lysis mediated by holin for progeny release^18^ termed as the “lysis from within” phenomenon. Structurally, lysins are categorized into either globular or modular. Most lysins earmarked for Gram-negative bacteria are globular, comprising a single Enzymatically Active Domain (EAD)^14,19^ while some are modular with two domains—the N-terminal EAD and the C-terminal Cell-wall Binding Domain (CBD)^20^. Though the EAD of lysins are highly conserved and categorized into muramidase, glucosaminidase, endopeptidase and l-alanine amidase, the CBD is variable and facilitates specific binding to bacteria cell wall^21^.

Enzymes like lysins are catalytic proteins whose efficiency and biological activity are determined by their interaction with inducted binding partners. Ideally, the molecular interactions of a Protein–Ligand duo are studied by arduous and expensive X-ray Crystallography or Nuclear Magnetic Resonance (NMR) methodology and the binding sites are pinpointed by techniques like mass-spectrometry. Alternatively, the interactions could be studied by computational modelling of Protein–Ligand complex through molecular docking studies to identify the binding-sites and estimate binding affinity by accurate scoring functions^22^. A notable study by Kemege et al.^23^ proved I-TASSER^24^ to be one of the most reliable structure prediction tools to accurately model three-Dimensional structure akin to high-resolution X-ray Crystallography and molecular docking studies well postulates the correlation of enzyme-ligand docking interactions with experimental bioactivity^25^.

**Figure 1:**
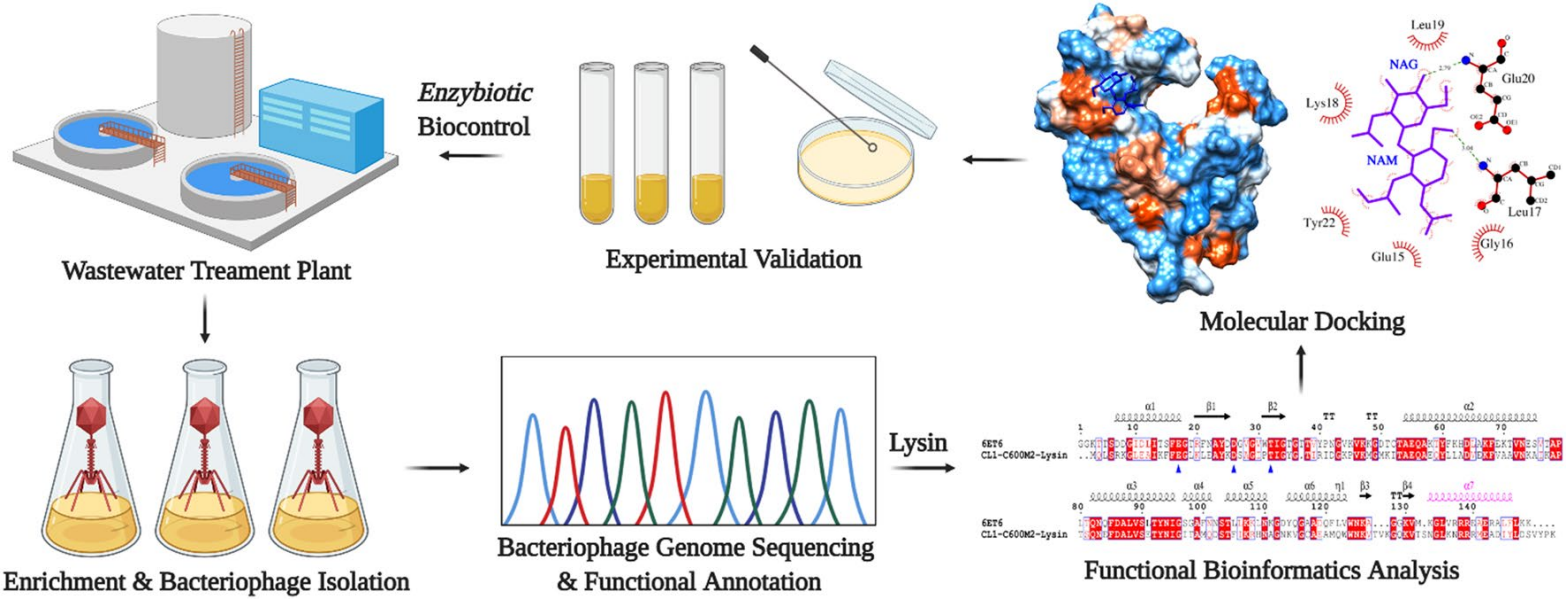
Summary of the research study. Wastewater samples were drawn from main wastewater treatment plant in Qatar, followed by enrichment and isolation of inherent bacteriophages, whole genome sequencing using Ion Torrent S5 next-generation sequencing (NGS) platform and functional annotation of the assembled bacteriophage genome. Gene products annotated as lysins were further analyzed for their potentiality as “enzybiotics” by in silico and experimental studies. This schematic representation was created with BioRender.com.

As summarized in Fig. 1, in this study, we propose the identical lysin integral to two novel bacteriophages isolated from wastewater^26^ as propitious “enzybiotic” with anti-bacterial potential by in silico analysis including molecular docking followed by experimental validation using turbidimetric reduction assay which may mark a new approach in the area of modern environmental biotechnology.

## Materials and methods

### Analysis of CL1-C600M2-lysin sequence

Lysin protein sequences derived from the genomes of *Escherichia* Phage C600M2 (Protein ID: UCJ01465) and *Escherichia* Phage CL1 (Protein ID: UCJ01321) were identical (Supplementary Fig. S1). Both the lysin protein sequences would hence be collectively termed as CL1-C600M2-lysin hereafter. Domain Analysis of CL1-C600M2-lysin was done using Conserved Domain Database (CDD)^27^. Further, the protein sequence of CL1-C600M2-lysin was subjected to protein-blast (accessed on 13 October 2021) against Refseq_Protein database to identify homologous proteins. Protein sequences sharing sequence identity > 70% and low E-value are 90% probable to share functional similarity^28^. Hence, such phage lysin hits sharing 100% query coverage were chosen, resulting in 61 protein entries with identity ranging from 85% to 98% (Supplementary Table 1). The accommodating environment of these lysin embodying phages were obtained from their respective GenBank records and publications.

### Phylogenetic tree of closely related phage lysin sequences

The retrieved lysin protein sequences from “Analysis of CL1-C600M2-lysin sequence” were aligned with CL1-C600M2-lysin using MUSCLE Alignment tool^29^. The best-fit protein substitution model JTT + G4 was estimated based on the Bayesian Information Criterion (BIC) using ModelTest-NG^30^. Then Maximim Likelihood (ML) Phylogenetic tree was generated using IQ-TREE^31^ by first creating 1000 ultrafast bootstraps (UFBoot)^32^ to minimize overestimation of bootstrap support (-bnni) and minimum correlation coefficient (-bcor) for UFBoot convergence criterion. Secondly, Shimodaira-Hasegawa like approximate likelihood ratio test (SH-alRT) with 1000 replicates was performed on the consensus tree derived from the previous run. The Standard bootstrap support (SBS) values for the ML analysis was estimated by concatenation of the generated bootstrap trees after 100 iterations with same alignment and substitution model mentioned above. A consensus tree using the original MUSCLE alignment input file was created. The support values UF/SH-aLRT/SBS were mapped to the ML tree and further annotated using Interactive Tree Of Life (iTOL) online tool^33^.

### Comparative analysis of phage proteome

NCBI Batch Entrez tool^34^ was used to download the complete proteomes of the 61 bacteriophages with their lysin gene product 85–98% identical to CL1-C600M2-lysin encoded in their genomes. The protein sequence dataset thus derived, along-with proteomes of *Escherichia* Phage CL1 and *Escherichia* Phage C600M2 was subjected to clustering based on sensitive search of sequences. There were 8105 protein sequences in total and those sharing at-least 80% identity and 100% query coverage were clustered using MMseqs2 tool^35^ to identify orthologous proteins in the protein dataset.

### Docking study of CL1-C600M2-lysin

Structure prediction of the CL1-C600M2-lysin protein sequence was done using I-TASSER protein modelling server^24^ with default settings. The model with the best C-Score was selected and validated with PROCHECK^36^ and assessed in ProSA^37^ prior molecular docking with prominent bacterial cell wall sugar receptors from literature^38^. The chemical entities analogous to bacterial cell wall receptors were identified from published studies and extracted from Protein Data Bank (PDB) as detailed in Table S1. The docking studies were performed using MTiAutoDock webserver^22^ in blind docking mode. The interacting residues were identified using LigPlot V.2.2^39^ and PDBsum^40^ and visualized using Chimera^41^ and PyMOL (Schrödinger, LLC) visualization software. The docked protein–ligand complexes were also visualized by embedding the ConSurf^42,43^ output derived by using the protein model of CL1-C600M2-lysin and the multiple sequence MUSCLE alignment (“Phylogenetic tree of closely related phage lysin sequences”) as input.

### Experimental validation for lytic activity of the CL1-C600M2-lysin

#### Plasmid construction and cloning

CL1-C600M2-lysin coding sequence was chemically synthesized (Integrated DNA Technologies) and cloned into the pRSET-emGFP expression vector (ThermoFisher Scientific) using BamH1 and EcoR1 restriction sites. This incorporated a N-terminal polyhistidine tag (His_6_). A stop codon was incorporated prior to the emGFP tag at the C-terminal resulting in CL1-C600M2-lysin with His Tag of molecular weight 21.269 KDa. Successful cloning was verified using PCR (Supplemental Figure S4).

#### Expression of lysin and protein purification

Transformed *Escherichia coli* BL21 (DE3) pLysS were grown in Luria broth supplemented with ampicillin and chloramphenicol media in specialized Erlenmeyer flasks to an OD of 70 Klett units using a Klett colorimeter. These cultures were induced with 1 mM isopropyl β-d-1-thiogalactopyranoside (IPTG) and shaken at 37 °C for 2.5 h at 225 rpm. Next, the bacteria were pelleted at 5000×*g* for 5 min and resuspended in 4 ml B-PER reagent (ThermoFisher Scientific) containing 1 × protease inhibitor cocktail per gram cell pellet. Following incubation at room temperature for 10 min, the suspension was homogenized by sonicating 20 s, followed a 1-min rest on ice which is repeated for a total 4 bursts. The lysate was centrifuged at 15,000×*g* for 5 min at 4 °C to separate the soluble proteins from the insoluble proteins. Ni–NTA chromatography was used to purify the his-tagged CL1-C600M2-lysin from the soluble proteins fraction. Proteins were eluted from the His-Bind Resin, Nickel charged (Novagen, EMB Biosciences, Darmstadt, Germany) using 6 volumes of elution buffer composed of 1 M imidazole, 0.5 M NaCl, 20 mM Tris–HCl, pH 7.9)**.** The imidazole-eluted fractions that contains the recombinant CL1-C600M2-lysin (as verified by SDS-PAGE and Western blot, Supplemental Figure S5), were combined at and dialyzed for overnight at 4 °C in deionized water containing 0.1% Trifluoroacetic acid (TFA) and 5 mM Dithiothreitol (DTT)**.** Purified CL1-C600M2-lysin was stored at − 20 °C until further analysis.

#### Turbidity reduction assay

Turbidity reduction assay was carried out according to Vander Elst et al.^44^ with some modifications. Briefly, *Escherichia coli* C, C600 (K-12), HB101 and B/R strains were grown in separate tubes overnight in Luria Broth at 37 °C in the air shaker. The cells were pelleted and washed with PBS and resuspended with a 1:1 mixture with purified CL1-C600M2-lysin to OD_600_ = 2. The OD_600_ was measured every 15 s at 37 °C for 1 h, shaking the 96-well plate between each measurement in the Varioskan™ LUX multimode microplate reader (ThermoScientific).

## Results

### Domain analysis of CL1-C600M2-lysin

The CL1-C600M2-lysin was interrogated in silico to set the precedents prior experimental confirmation for the possibility to be an “enzybiotic”. The lysin had intrinsic domains-“cd00737: endolysin_autolysin” with no CBD, suggesting it to be a globular *N*-acetyl-muramidase^14,19,20^. A search for globular endolysins in Protein Data Bank (PDB) yielded entry 6ET6^45^ as the only X-ray crystal structure of endolysin AcLys classified as *N*-acetyl-muramidase antimicrobial protein encoded in genome of *Acinetobacter baumannii* AB 5075.

**Figure 2:**
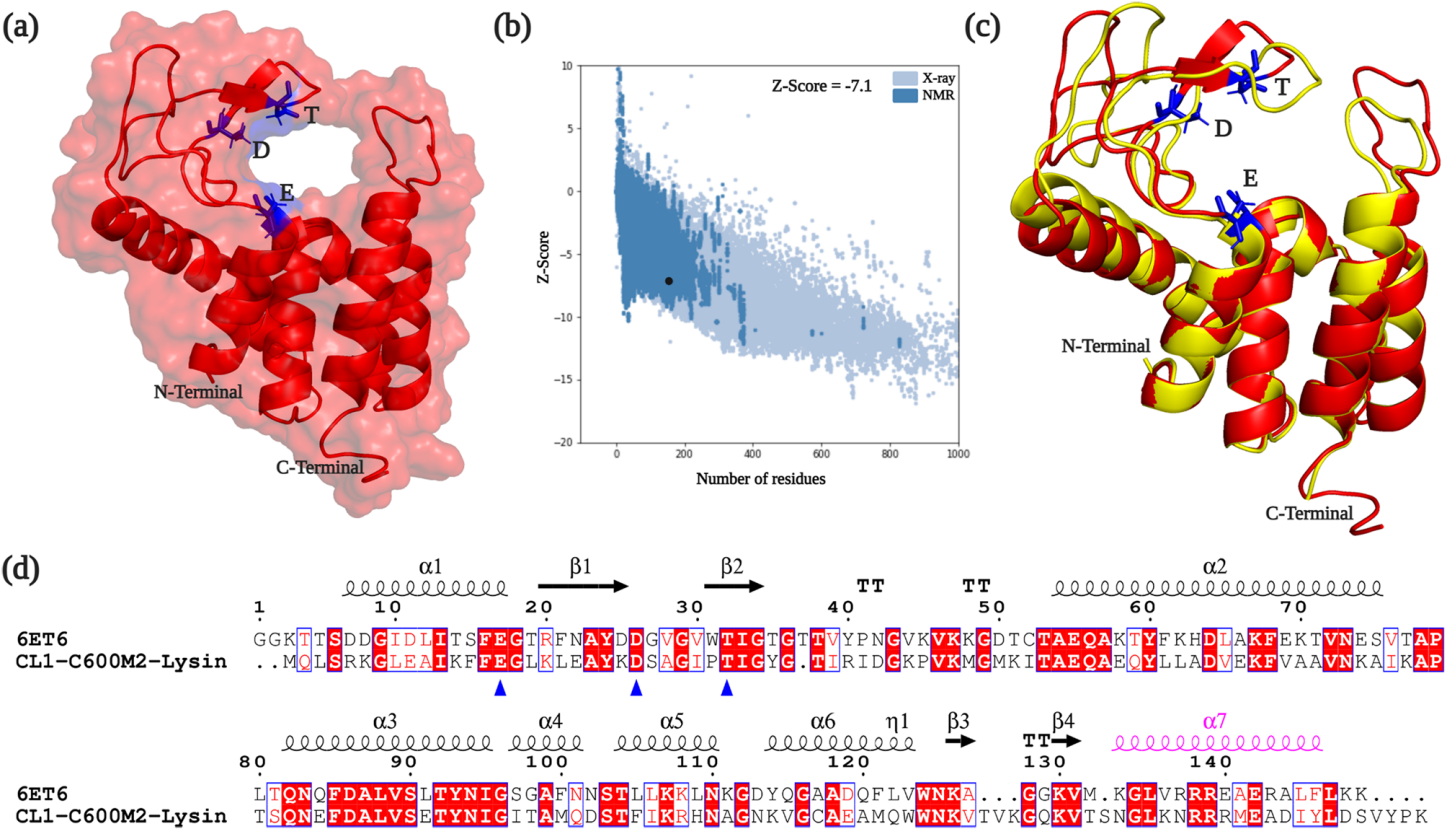
Sequential and Structural analysis: (**a**) three-dimensional CL1-C600M2-lysin modelled structure predicted by I-TASSER with labelled N and C-terminal region along with the catalytic triad Glu(E)-Asp(D)-Thr(T) represented as blue sticks. (**b**) Z-score plot generated by ProSA for predicted model quality assessment. (**c**) Superposition of CL1-C600M2-lysin (Red) with AcLys (PDB Code: 6ET6, Yellow). The catalytic triad is represented as blue sticks and labelled. (**d**) Multiple sequence alignment of AcLys (PDB Code: 6ET6) and CL1-C600M2-lysin. The catalytic triad is marked by blue triangles and the C-terminal α-helix enriched with positively charged residues is highlighted in Pink.

Structure of CL1-C600M2-Lysin was modelled using I-TASSER webserver (Fig. 2a), validated with PROCHECK^36^ (Supplementary Fig. S2) and Quality-assessed using ProSA^37^ (Fig. 2b) followed by structural comparison with 6ET6 (Fig. 2c). Z-Score of CL1-C600M2-lysin was -7.1 (Fig. 2b), well within the range of experimentally published structures. CL1-C600M2-lysin and 6ET6 have conserved Glu-Asp-Thr catalytic triad as represented in Fig. 2d. Superposition of the CL1-C600M2-lysin structure with PDB:6ET6 (Fig. 2c) revealed close similarity with RMSD between corresponding Cα-atoms of 0.46 Å. C-terminal α-helix of AcLys, enriched with positively-charged residues significantly facilitates cell-wall lysis^45^. Similar domain architecture could be observed in the CL1-C600M2-lysin as well (Fig. 2d).

### Phylogenetic tree analysis

**Figure 3:**
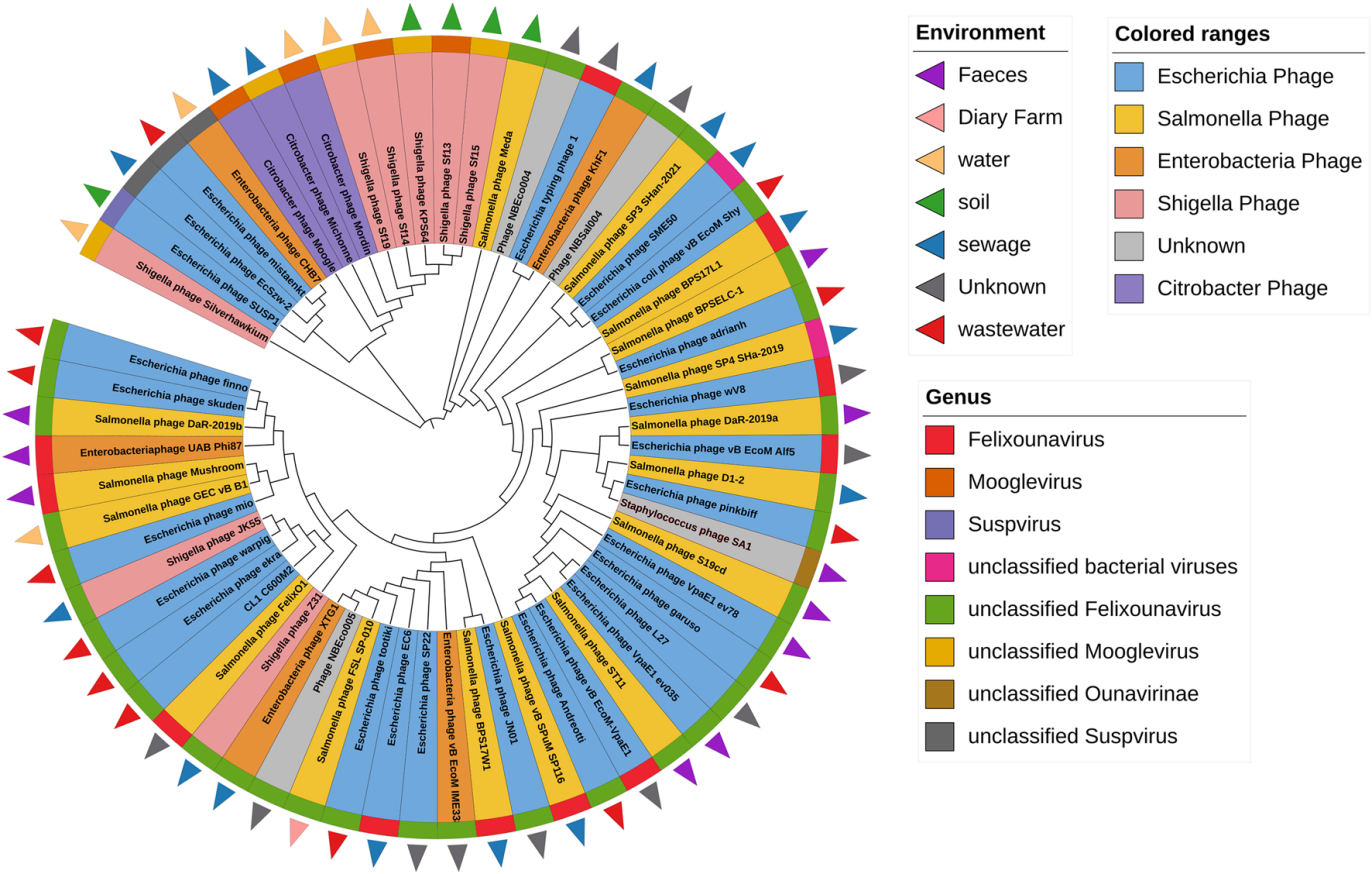
ML phylogenetic tree: 61 highly identical phage lysin protein sequences sharing 100% query coverage were derived by querying CL1-C600M2-lysin in Protein Blast tool against Refseq_Protein database. Colored triangles represent the generalized isolation source of the Phage as detailed in the legend titled Environment. Colored ranges indicate the host organism of the Phage. The outer circle of the ML Tree is colored respective to the Genus of the Phage.

The CL1-C600M2-lysin sequence was subjected to protein-blast against Refseq_Protein database to identify homologous proteins. 61 Phage protein sequences annotated as either lysin, endolysin or putative lysin with 100% query coverage and sharing 85–98% sequence identity was determined as listed in Supplementary Table 1 and summarized in Table S2. A ML phylogenetic tree was estimated from the derived lysin sequences to discern their relatedness (Fig. 3). Apart from wastewater, these lysins were inherent in phages thriving in various similar environments like sewage, fecal samples, soil from poultry and animal farms etc. Among the 49 bacteriophages with eminent information about isolation source, 27 (55%) were native to sewage or wastewater. This clearly indicates the involvement of CL1-C600M2-lysin in host–pathogen interactions endemic to wastewater environment.

Moreover, it can be observed from the ML tree (Fig. 3) that lysins inherent in bacteriophages—*Shigella phage Z31, Salmonella phage FelixO1, Escherichia phage ekra, Escherichia phage warpig, Shigella phage JK55, Escherichia phage mio, Salmonella phage GEC_vB_B1, Salmonella phage Mushroom, Enterobacteriaphage UAB_Phi87, Salmonella phage DaR-2019b, Escherichia phage skuden, Escherichia phage finno* cluster well with CL1-C600M2-lysin. These lysin sequences share identity ranging from 98% to 96%. Since, lysins sharing high similarity with CL1-C600M2-lysin were involved in host–pathogen interactions of multiple species in the *Enterobacteriaceae* family, this could indicate the potency of CL1-C600M2-lysin against multiple hosts.

Interestingly, lysin from *Staphylococcus phage* SA1 shares 96% identity with CL1-C600M2-lysin. It has been published that *Staphylococcus phage* SA1 is effective against infection by *Staphylococcus,* a Gram-Positive bacteria in animal models^46^. This could further suggest the effectiveness of CL1-C600M2-lysin against both Gram-positive and Gram-negative bacteria. Further, the affinity of CL1-C600M2-lysin to bacterial cell wall receptors was predicted using molecular docking studies.

### Clustering analysis of phage proteome

A total of 8105 protein sequences were retrieved as described in “Comparative analysis of phage proteome” for sensitive-search clustering analysis using MMseqs2 tool^35^. The protein sequences clustered into 797 independent clusters, such that the sequences in each cluster share at least 80% identity with 100% coverage (listed in Supplementary Table 2). There were about 368 unique clusters (46%) with only a single sequence and around 369 clusters with number of sequences ranging between 2 and 49, leaving only 60 clusters constituted by at least 50 proteins. In other words, only 60 clusters had orthologous proteins from at least 50 candidate phage species (Table S3). The conserved protein clusters majorly consisted proteins involved in Structural architecture (baseplate assembly protein, head maturation protease, hypothetical protein/baseplate assembly protein, hypothetical protein/baseplate protein, hypothetical protein/putative membrane protein, hypothetical protein/putative portal protein, hypothetical protein/structural protein, hypothetical protein/tape measure chaperone, major capsid protein and tape measure chaperone) followed by nucleotide metabolism (ribonucleoside triphosphate reductase alpha/beta subunit, glutaredoxin, thymidylate synthase, dNMP kinase, anaerobic NTP reductase, ribose-phosphate pyrophosphokinase and dihydrofolate reductase), replication module (DNA ligase, DNA primase/helicase, exonuclease, homing endonuclease/NAD synthetase, Hypothetical protein/dsDNA binding protein and terminase large subunit), Lytic module (endolysin/lysin, holin-hypothetical or putative holin, Hypothetical protein/i-spanin and rIIB lysis inhibitor) and Hypothetical protein of unknown function. Among the 60 conserved protein clusters, 31 of them comprised of Hypothetical proteins of unknown function (50–63 proteins of the kind in each cluster) followed by 2 clusters of Baseplate assembly proteins (63 proteins of the kind in each cluster) and the rest were disjoint clusters (50–63 proteins of each kind in each cluster).

Further to it, search for phage host recognition proteins in the protein dataset, revealed the prevalence of 19 host recognition proteins (annotated as Tail Fiber Protein, Putative Tail Fiber Protein, Putative Tail Protein, Putative Tail Fiber Protein Gp37, Tail Sheath Protein, Putative Tail Tape Measure Chaperone, Hk97 Major Tail Subunit, Head–Tail Preconnector Protein, Side Tail Fiber Protein, Long Tail Fiber Protein, Conserved Tail Assembly Protein, Tail Tube Protein, Tail Protein, Putative Tail Tape Measure Protein, Tail Tape Measure Protein, Tail Assembly Protein, Tail Length Tape Measure Protein and Minor Tail Protein) clustered within 69 clusters (Table S3).

### Molecular docking study of CL1-C600M2-lysin

**Figure 4:**
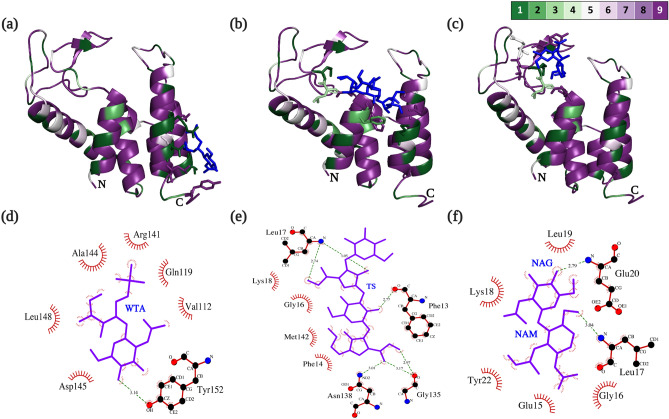
Protein–ligand docked complexes: the 3D structure of CL1-C600M2-lysin is represented using ConSurf^42,43^ output complexed with ligands (**a**) WTA; (**b**) TS and (**c**) PG. Interacting residues are denoted as sticks and colored based on the represented conservation scale with dark purple being highly conserved and dark green being highly variable. N and C terminal of each structure is indicated by N and C, respectively. All ligands are represented in dark blue irrespective of their kind. Ligplot maps for interacting residues for protein ligand complexes, (**d**) CL1-C600M2-lysin–WTA, (**e**) CL1-C600M2-lysin–TS and (**f**) CL1-C600M2-lysin–PG. The sugar components of PG, β-(1,4) linked *N*-acetylglucosamine (NAG) and *N*-acetylmuramic acid (NAM) are labelled.

It can be noted from Supplementary Fig. S3, and Fig. 4a,b that the residues of CL1-C600M2-lysin interacting with WTA and TS were highly conserved amongst similar phage lysin sequences derived from protein-blast analysis. It can be deduced from Fig. 4c that PG substrate binding residues are within the binding cavity surrounding the catalytic triad and mostly conserved.

Table 1 summarizes the interacting residues with each ligand and the corresponding binding affinity values. Sequential interacting amino acid residues could probably suggest an interacting motif in CL1-C600M2-lysin that facilitates binding to the corresponding ligand. In CL1-C600M2-lysin-WTA protein–ligand complex, the motif Arg141-Ala144–Asp145–Leu148–Tyr152 could be the motif involved in recognition of Gram- Positive bacterial cell wall with Tyr152 forming a hydrogen bond of length 3.14 Å (Fig. 4d). While in CL1-C600M2-lysin–TS complex, the motif Phe13–Phe14–Gly16–Leu17–Lys18 might be involved in recognition of Gram-negative bacterial cell wall with Phe13 (2.77 Å) and Leu17 (2.74 Å and 3.25 Å) forming hydrogen bonds (Fig. 4e). The dual purpose of motif Phe13–Phe14–Glu15–Gly16–Leu17–Lys18 and efficiency of CL1-C600M2-lysin against Gram-negative bacterial cell wall could be speculated from its interaction and hydrogen bond formation with both TS and PG (Fig. 4e,f).

**Table 1:** Summary of molecular docking analysis of CL1-C600M2-lysin with selected bacterial cell surface receptors as represented in Ligplot interaction map in Fig. 4d–f.

Cell wall receptor	Binding affinity (kcal/mol)	Interacting residues
WTA	− 10.67	Val112, Gln119, Arg141, Ala144, Asp145, Leu148, Tyr152*
TS	− 11.15	Phe13*, Phe14, Gly16, Leu17*, Lys18, Gly135*, Asn138*, Met142
PG	− 6.53	Glu15, Gly16, Leu17*, Lys18, Leu19, Glu20*, Tyr22

The binding affinity of all the protein–ligand partners were for the 1st ligand conformation as predicted by MTiAutoDock. Sequential amino acid residues are in bold.

*Residues forming hydrogen bond.

### Assessment of lytic activity of CL1-C600M2-lysin

**Figure 5:**
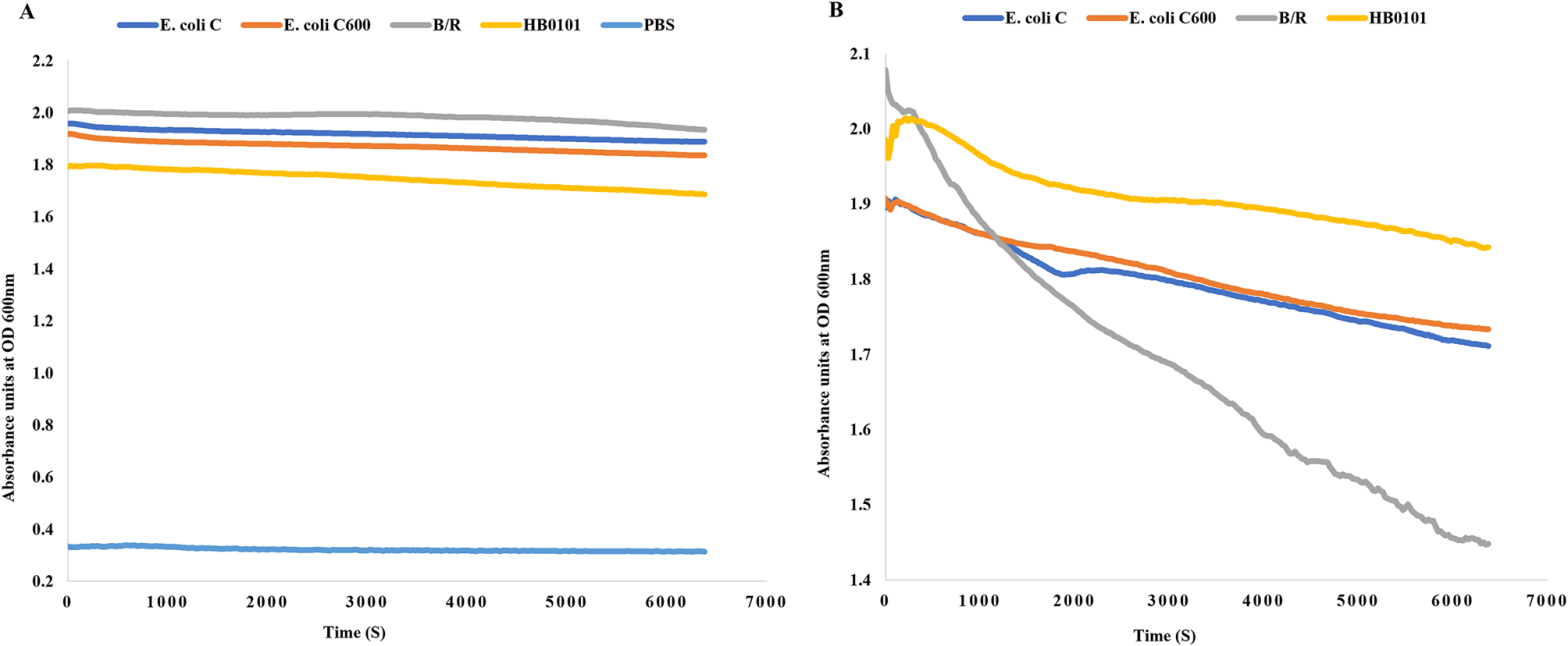
Turbidity reduction assays: (**A**) *Escherichia coli* strains C, C600, B/R and HB101 in PBS serve as negative controls. (**B**) The same *Escherichia coli* strains with 80 ng of purified CL1-C600M2-lysin.

To assess the lytic capacity of CL1-C600M2-lysin turbidity reduction assay was performed on *Escherichia coli* C, C600 (K-12), HB101 and B/R strains (Fig. 5) by mixing 80 ng of purified CL1-C600M2-lysin. The bacterial strains in PBS served as negative control for comparison. It can be well observed from Fig. 5B that the CL1-C600M2-lysin was most effective against *Escherichia coli* B/R followed by *Escherichia coli* C, C600 and HB101 strains. While the negative control does not show reduction in the OD values. Thus, the turbidity reduction assay using CL1-C600M2-lysin clearly predicates its utility as a possible anti-bacterial agent.

## Discussion

Phage infection machinery majorly consist of tail fibers, base plates and lysins. Host recognition occurs through a reversible interaction of the tip of the long tail fibers with bacterial outer-membrane (OM) components^47^. This activates the baseplate and binding of short tail fibers on cell-surface receptors, followed by contraction of the outer tail sheath and penetration of inner tail tube into cell-membrane and injection of the viral-DNA into the bacteria^48,49^. Each Phage–Host interaction is attuned with great specificity^50,51^.

### In-silico analysis of endolysins

Endolysins are bifunctional enzymes marshalling precise binding to bacterial-receptor and enzymatic hydrolysis of its cell-wall which is exhorted by crucial domains. The endolysins of most phages that infect gram-negative bacteria, comprise a single catalytic domain, whereas gram-positive bacterial phages embody N-terminal EAD and C-terminal CBD. The location and sizes of the domains are known to vary. The CBD and its appurtenant binding site eventually dictate the endolysin’s substrate-affinity and therefore robust bacterial infection^52^.

In consensus, endolysins such as AcLys and PlyE146 categorized as muramidases and tested for their anti-microbial ability against pathogens *A. baumannii, E. coli, P. aeruginosa, K. pneumoniae* and *S. enterica* have N-terminal “cd00737: endolysin_autolysin” domain and a highly positive-charged stretch of amino acids at their C-Terminal^45,53^. This domain architecture highly correlates with CL1-C600M2-lysin accentuating it as promising enzybiotic candidate. Protein-Blast of CL1-C600M2-lysin (Fig. 3) corroborates the likelihood of broad-range of target pathogens like *Enterobacteria, Escherichia, Shigella* and *Salmonella*.

A study by Grose et al.^54^ classifies bacteriophages into 56 clusters inclusive of 32 lytic and 24 temperate clusters based on available genomic sequence data. Inter-cluster members share genomic similarity but significant dissimilarity intra-cluster. It could be inferred from Fig. 3 that the CL1-C600M2-lysin corresponds to the lytic cluster “Felix-O1-like” it is 99% identical with *Salmonella phage felix-O1*. This particular lysin is widely proven to target majority of Salmonella family and employed in effective biocontrol of pathogenic organisms^55,56^. Lysin of *Escherichia phage* vB_EcoM_VpaE1 with host range of VpaE1 E. coli B strains like BE, BL21, BL21(DE3), B40, BE-BS^57^ also shares 98% identity with CL1-C600M2-lysin. *Salmonella phage Mushroom* is a constituent of IntestiPhage, a combination of 23 phages capable to infect a range of enterobacteria strains^58^ while *Samonella Phage vB SPuM SP116* is capable of infecting 9 serotypes of *Salmonella*, namely, *Pullorum*, *Enteritidis*, *Indiana*, *Typhimurium*, *Infantis*, *Montevideo Heidelberg*, *Paratyphi A*, and *Derby*. Both the afore-mentioned phages share 96% and 97% identity with the CL1-C600M2-lysin, respectively. Interestingly, the research study by Low et al.^59^ correlates net-positive charge of the catalytic domain of lysins with bactericidal efficiency. CL1-C600M2-lysin has a net charge of + 6.1 at Ph-7.0 (using http://protcalc.sourceforge.net/), upholding it to be a potential “enzybiotic”. Further in silico analysis through clustering of Proteome from wastewater-endemic phages, molecular docking studies, and experimental validation has suggested the same.

### Analysis of proteome from wastewater-endemic phages

**Figure 6:**
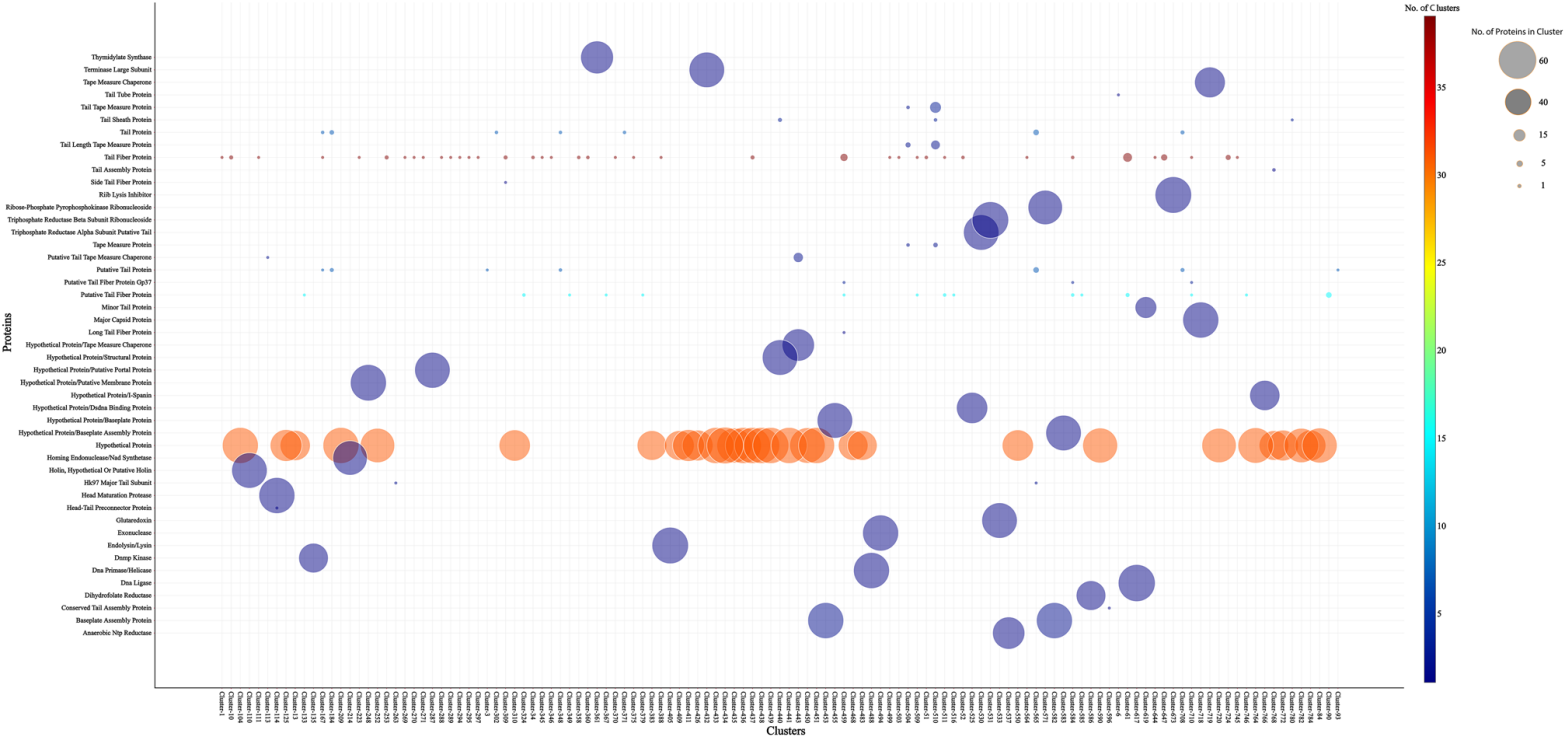
Bubble plot of crucial protein clusters. Crucial proteins clusters distilled by clustering of Phage proteome are represented as bubbles. Color of bubble represents the number of clusters that contain the protein kind, ranging from dark brown to dark blue. And the size of the bubble represents the quantity of the protein kind within the cluster.

Systematic clustering analysis of the protein dataset derived from proteome of 61 phages, *Escherichia phage CL1 and Escherichia phage C600M2* was done to unravel their inherent biodiversity in spite of likeness of their lysin proteins. The two major cluster sets of interest were the conserved clusters and the host-recognition protein clusters. It can be deciphered from Fig. 6 that majority of the host recognition proteins of the candidate phages were grouped into multiple clusters, especially the Tail fiber proteins (39 clusters, quantity: 1–12), Putative Tail fiber proteins (15 clusters; quantity: 1–6), Putative Tail protein (7 clusters; quantity: 1–6) and Tail proteins (7 clusters; quantity: 2–6). Interestingly, all the 35 minor tail proteins in the dataset clustered into a disjoint cluster.

Typically, phage–host adsorption is a two-step process involving preliminary reversible attachment to the host cell receptors followed by irreversible attachment either by strengthening the initial bond or by binding to secondary receptors. Distal phage tail elements like Tail fibers form reversible interactions with exposed and highly accessible host cell wall components while irreversible interactions with secondary host-receptors are facilitated by short/minor tail proteins^60^. The tail structures of bacteriophages are the key determinants of host specificity and the observation of diverse distal tail protein elements despite a highly conserved lysin among these bacteriophages endemic to wastewater could indicate the potency of CL1-C600M2-lysin against multiple bacterial species.

### Molecular docking studies

Phage lysins typically recognize varied bacterial cell-wall receptors depending on their host range. However, due to insufficient research on designated ligands, it can be presumed that lysins mostly target cell wall carbohydrates and the specificity of its cell wall binding region determines its range of target organisms^52^. WTA and LPS are one of unique cell surface ligands that distinguish between gram-positive and gram-negative bacteria. Minimal repeating glyco-polymers of the above-mentioned ligands used in similar experimental initiatives were used in molecular docking studies with CL1-C600M2-lysin to identify propitious cell wall binding motifs. The synthetic PG analog used in the study of T4 phage lysozyme’s enzyme–substrate interactions was also implemented in docking studies to computationally model and interrogate substrate affinity of CL1-C600M2-lysin.

With reference to Fig. S3, both the N-terminal catalytic and C-Terminal cell-wall binding regions of CL1-C600M2-lysin are well conserved among the lysins from bacteriophages isolated from wastewater and similar environments (Table S2). Probably suggesting multi-host versatility of its cell-wall binding region and proving to be the right candidate to designate as an "enzybiotic" bio-control agent for wastewater treatment purposes.

**Figure 7:**
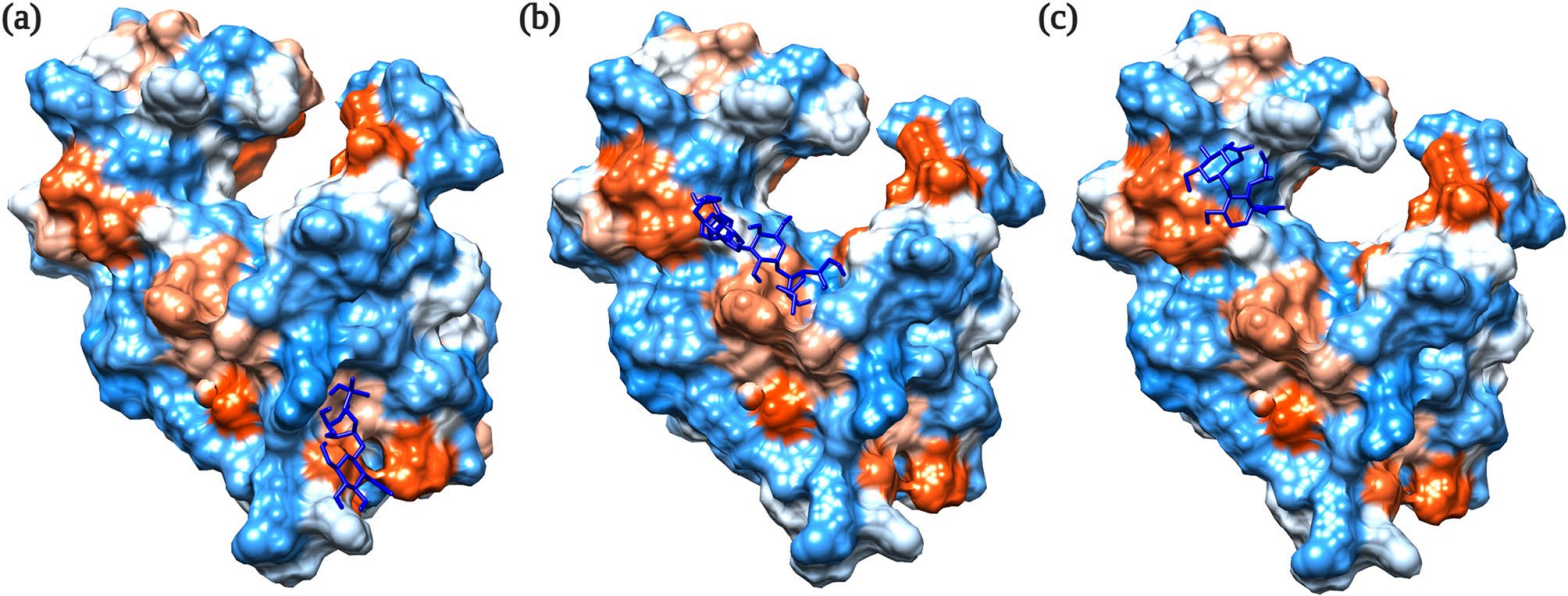
Hydrophobicity of protein–ligand complexes. The 3-dimensional structure of CL1-C600M2-lysin is represented using the hydrophobicity attribute in Chimera, complexed with ligands (**a**) WTA; (**b**) TS and (**c**) PG. The protein surface is colored based on hydrophobiciy of the underlying residues ranging from blue, being highly hydrophilic to red, being highly hydrophobic. All ligands are represented in dark blue irrespective of their kind.

Adding to it, most interacting residues of CL1-C600M2-lysin irrespective of the ligand were hydrophobic (Fig. 7). Corroborating the study by Yan et al.^61^, residues in proximity of predicted cell-wall binding motifs of CL1-C600M2-lysin could be modified to be more hydrophobic for improved efficiency for exogenous application.

### Antimicrobial activity of CL1-C600M2-lysin

The turbidity reduction assay was used to demonstrate the antimicrobial activity of CL1-

C600M2-lysin in several *Escherichia coli* strains. The decrease in turbidity in a bacterial solution is an indirect measure of cell death. The effect of factors such as buffer components and osmotic pressure changes are accounted for in the bacteria and PBS controls (Fig. 5A). Therefore, the turbidity reduction observed in the four *Escherichia coli* strains in the presence of CL1-C600M2-lysin is indicative of the possible “enzybiotic” nature of the lysin and corroborates with the bioinformatics analysis.

Overall, we present comprehensive functional bioinformatics analysis and experimental validation of identical lysin gene products (collectively termed as CL1-C600M2-lysin) identified from two novel *Myoviridae* bacteriophages, *Escherichia* Phage C600M2 and *Escherichia* Phage CL1, which were isolated from wastewater treatment plant in the State of Qatar. CL1-C600M2-lysin was analyzed in-silico to gain insights for their practicability as prospective “enzybiotics” for water-treatment and set the necessary precedents prior experimental venture. Followed by experimental assessment of the lytic activity of CL1-C600M2-lysin using turbidimetric reduction assay.

## Conclusions

In summary, by using efficient computational strategies of comparative sequence analysis, proteome clustering, protein structure modelling, protein structural analysis and molecular docking studies we present complete in silico characterization of identical lysin, CL1-C600M2-lysin, from two bacteriophages isolated from the wastewater samples collected from a treatment plant in State of Qatar. Further experimental investigation of detrimentality of CL1-C600M2-lysin towards laboratory strains of *Escherichia coli* revealed the lysin to be most lethal towards *Escherichia coli* B/R followed by *Escherichia coli* C, C600 and HB101 strains.

Encompassing comprehensive computational characterization of CL1-C600M2-lysin to set the essential premises for substantial experimentation withal experimental confirmation of the lysin’s utility as a potential “enzybiotic”, this study presents a novel amalgamation of research strategies to corroborate prospectiveness of CL1-C600M2-lysin to be purposed as a biocontrol agent distinctive to wastewater environment.

Given the necessity to preserve scarce water resources, reclamation of treated wastewater is an essential step towards water security. CL1-C600M2-lysin can potentially be used as an efficient “enzybiotic” to biocontrol wastewater endemic bacteria. CL1-C600M2-lysin could eradicate the residual pathogenic bacteria still persistent in treated wastewater to assure safety and quality-control prior recycling. Moreover, research inquiry for the therapeutic potential of CL1-C600M2-lysin could further widen the scope of this study.

## Supplementary Information

Supplementary Information 1.

Supplementary Information 2.

Supplementary Information 3.

## Data Availability

*Escherichia* Phage C600M2 (GenBank accession: OK040807.1) and Lysin (GenBank accession: UCJ01465.1) Phage Phage CL1 (GenBank accession: OK040806.1) and Lysin (GenBank accession: UCJ01321.1).
