# Supplementary material for: Substantiation of propitious “Enzybiotic” from two novel bacteriophages isolated from a wastewater treatment plant in Qatar
Source: Sci Rep. 2022 May 31;12:9093. doi: 10.1038/s41598-022-13171-8 (PMC9156722; doi:10.1038/s41598-022-13171-8)
Supplement: Supplementary file 1 — Supplementary Information 1. [file 41598_2022_13171_MOESM1_ESM.pdf]

Supplementary Information

Substantiation of Propitious "Enzybiotic" from two Novel Bacteriophages Isolated from a Wastewater Treatment plant in Qatar

Ramya Ramadoss[1], Moza Al-Shukri[2], Basem Shomar[3], Valentin Alekseevich Ilyin[4], Annette Shoba Vincent[5*]

[1]Biological Sciences, Carnegie Mellon University Qatar, PO box 24866, Doha, Qatar.

Email: rramado2@andrew.cmu.edu

[2] Biological Sciences, Carnegie Mellon University Qatar, PO box 24866, Doha, Qatar.

Email: mshukri@andrew.cmu.edu

[3] Environmental Science Center (ESC), Qatar University, PO box 2713, Doha, Qatar.

Email: bshomar@qu.edu.qa

[4]Computational Biology, Carnegie Mellon University Qatar, PO box 24866, Doha, Qatar.

Email: valentin.ilyin@gmail.com

[5]Biological Sciences, Carnegie Mellon University Qatar, PO box 24866, Doha, Qatar

Email: annettev@andrew.cmu.edu, Tel: +974 4484852

[*]**Corresponding Author**

Table S1: List of candidate chemical entities chosen as ligands for molecular docking studies with CL1-C600M2-Lysin.

**PDB entry and PubChem CID:**	**Description**
5D6C<br>92044073	Minimal repeat of synthetic Wall Teichoic Acid (WTA) used in study for effect of WTA on anti-*S. aureus* antibody [1]
5LSH<br>129009673	Minimal repeat of Tetrasaccharides (TS) representing O-Chain of *Klebsiella pneumoniae O1* LPS used to study its interactions with Human Lysozyme. [2]
148L	Minimal repeat of peptidoglycan (PG) used to study the structural aspects of enzyme substrate interactions of mutant T4 lysozyme. [3]

Table S2: Summarized list of lysin sequences in Supplementary Table 1 file. The lysin sequences share sequence identity >85% and have 100% query coverage. Genus column has the Genus and Rank of the source bacteriophage in bold and in italics respectively. Environment column represents the generalized isolation source of the organism as submitted in their GenBank entry. Total count column consolidates the count of entries for each row category. Columns highlighted in orange are the predominant Rank and Environment.

Genus	Environment						Total Count
	Faeces	Sewage	Soil	Unknown	Wastewater	Water	
**Felixounavirus**	**10**	**10**	**1**	**12**	**11**	**1**	**45**
Escherichia virus Alf5				1			**1**
Escherichia virus EC6		1					**1**
Escherichia virus TP1				1			**1**
Escherichia virus VpaE1				1			**1**
Escherichia virus wV8				1			**1**
Salmonella virus BPS17L1		1					**1**
Salmonella virus BPS17W1		1					**1**
Salmonella virus FelixO1				1			**1**
Salmonella virus Mushroom	1						**1**
Salmonella virus SP116		1					**1**
Salmonella virus UAB87	1						**1**
unclassified Felixounavirus	8	6	1	7	11	1	**34**
**Mooglevirus**		**2**	**1**			**6**	**9**
Citrobacter virus Moogle		1					**1**
Citrobacter virus Mordin						1	**1**
Shigella virus Sf13						1	**1**
Shigella virus Sf14						1	**1**
unclassified Mooglevirus		1	1			3	**5**
**Suspvirus**		**1**	**1**		**1**	**1**	**4**
Escherichia virus SUSP1			1				**1**
unclassified Suspvirus		1			1	1	**3**
**unclassified Ounavirinae**	**1**						**1**
**unclassified bacterial viruses**		**2**					**2**
**Total Count**	**11**	**15**	**3**	**12**	**12**	**8**	**61**

Table S3: Summarized list of clustered significant proteins from Supplementary Table 2 file:

30 Conserved protein kinds clustered within 60 clusters (Highlighted in Green) and 19 Host recognition protein kinds clustered within 69 clusters (Highlighted in Orange) summarized from the 8105 protein sequences in Supplementary Table 2.

Cluster	Protein Description	No. of Proteins in Cluster
Cluster-1	Tail Fiber Protein	1
Cluster-111	Tail Fiber Protein	1
Cluster-113	Putative Tail Tape Measure Chaperone	1
Cluster-114	Head-Tail Preconnector Protein	1
Cluster-133	Putative Tail Fiber Protein	1
Cluster-167	Putative Tail Protein	1
Cluster-167	Tail Fiber Protein	1
Cluster-223	Tail Fiber Protein	1
Cluster-263	Hk97 Major Tail Subunit	1
Cluster-269	Tail Fiber Protein	1
Cluster-270	Tail Fiber Protein	1
Cluster-271	Tail Fiber Protein	1
Cluster-288	Tail Fiber Protein	1
Cluster-289	Tail Fiber Protein	1
Cluster-294	Tail Fiber Protein	1
Cluster-295	Tail Fiber Protein	1
Cluster-297	Tail Fiber Protein	1
Cluster-3	Putative Tail Protein	1
Cluster-309	Side Tail Fiber Protein	1
Cluster-345	Tail Fiber Protein	1
Cluster-346	Tail Fiber Protein	1
Cluster-349	Putative Tail Fiber Protein	1
Cluster-367	Putative Tail Fiber Protein	1
Cluster-370	Tail Fiber Protein	1
Cluster-375	Tail Fiber Protein	1
Cluster-379	Putative Tail Fiber Protein	1
Cluster-388	Tail Fiber Protein	1
Cluster-459	Long Tail Fiber Protein	1
Cluster-459	Putative Tail Fiber Protein	1
Cluster-459	Putative Tail Fiber Protein Gp37	1
Cluster-499	Tail Fiber Protein	1
Cluster-503	Tail Fiber Protein	1

Cluster-509	Putative Tail Fiber Protein	1
Cluster-509	Tail Fiber Protein	1
Cluster-511	Tail Fiber Protein	1
Cluster-516	Putative Tail Fiber Protein	1
Cluster-564	Tail Fiber Protein	1
Cluster-565	Hk97 Major Tail Subunit	1
Cluster-584	Putative Tail Fiber Protein Gp37	1
Cluster-585	Putative Tail Fiber Protein	1
Cluster-596	Conserved Tail Assembly Protein	1
Cluster-6	Tail Tube Protein	1
Cluster-644	Tail Fiber Protein	1
Cluster-710	Putative Tail Fiber Protein	1
Cluster-710	Putative Tail Fiber Protein Gp37	1
Cluster-710	Tail Fiber Protein	1
Cluster-745	Tail Fiber Protein	1
Cluster-746	Putative Tail Fiber Protein	1
Cluster-780	Tail Sheath Protein	1
Cluster-93	Putative Tail Protein	1
Cluster-167	Tail Protein	2
Cluster-302	Tail Protein	2
Cluster-324	Putative Tail Fiber Protein	2
Cluster-34	Tail Fiber Protein	2
Cluster-348	Putative Tail Protein	2
Cluster-348	Tail Protein	2
Cluster-358	Tail Fiber Protein	2
Cluster-360	Tail Fiber Protein	2
Cluster-371	Tail Protein	2
Cluster-504	Putative Tail Tape Measure Protein	2
Cluster-504	Tail Tape Measure Protein	2
Cluster-51	Tail Fiber Protein	2
Cluster-510	Tail Sheath Protein	2
Cluster-511	Putative Tail Fiber Protein	2
Cluster-52	Tail Fiber Protein	2
Cluster-584	Putative Tail Fiber Protein	2
Cluster-584	Tail Fiber Protein	2
Cluster-768	Tail Assembly Protein	2
Cluster-10	Tail Fiber Protein	3
Cluster-184	Putative Tail Protein	3
Cluster-253	Tail Fiber Protein	3
Cluster-309	Tail Fiber Protein	3
Cluster-437	Tail Fiber Protein	3

Cluster-440	Tail Sheath Protein	3
Cluster-61	Putative Tail Fiber Protein	3
Cluster-708	Putative Tail Protein	3
Cluster-708	Tail Protein	3
Cluster-184	Tail Protein	4
Cluster-510	Putative Tail Tape Measure Protein	4
Cluster-504	Tail Length Tape Measure Protein	5
Cluster-724	Tail Fiber Protein	5
Cluster-565	Putative Tail Protein	6
Cluster-565	Tail Protein	6
Cluster-90	Putative Tail Fiber Protein	6
Cluster-647	Tail Fiber Protein	7
Cluster-459	Tail Fiber Protein	9
Cluster-510	Tail Length Tape Measure Protein	12
Cluster-61	Tail Fiber Protein	12
Cluster-443	Putative Tail Tape Measure Chaperone	13
Cluster-510	Tail Tape Measure Protein	16
Cluster-619	Minor Tail Protein	35
Cluster-135	dNMP Kinase	50
Cluster-383	Hypothetical Protein	50
Cluster-409	Hypothetical Protein	50
Cluster-468	Hypothetical Protein	50
Cluster-483	Hypothetical Protein	50
Cluster-586	Dihydrofolate Reductase	50
Cluster-768	Hypothetical Protein	50
Cluster-13	Hypothetical Protein	51
Cluster-766	Hypothetical Protein/I-Spanin	51
Cluster-426	Hypothetical Protein	52
Cluster-719	Tape Measure Chaperone	52
Cluster-772	Hypothetical Protein	52
Cluster-784	Hypothetical Protein	52
Cluster-310	Hypothetical Protein	53
Cluster-525	Hypothetical Protein/dsDNA Binding Protein	53
Cluster-550	Hypothetical Protein	53
Cluster-125	Hypothetical Protein	54
Cluster-411	Hypothetical Protein	54
Cluster-443	Hypothetical Protein/Tape Measure Chaperone	55
Cluster-537	Anaerobic NTP Reductase	55
Cluster-361	Thymidylate Synthase	56
Cluster-214	Homing Endonuclease/NAD Synthetase	59
Cluster-252	Hypothetical Protein	59

Cluster-571	Ribose-Phosphate Pyrophosphokinase	59
Cluster-720	Hypothetical Protein	59
Cluster-782	Hypothetical Protein	59
Cluster-435	Hypothetical Protein	60
Cluster-590	Hypothetical Protein	60
Cluster-84	Hypothetical Protein	60
Cluster-110	Holin, Hypothetical or Putative Holin	61
Cluster-287	Hypothetical Protein/Putative Portal Protein	61
Cluster-432	Terminase Large Subunit	61
Cluster-440	Hypothetical Protein/Structural Protein	61
Cluster-450	Hypothetical Protein	61
Cluster-455	Hypothetical Protein/Baseplate Protein	61
Cluster-530	Ribonucleoside Triphosphate Reductase Alpha Subunit	61
Cluster-533	Glutaredoxin	61
Cluster-583	Hypothetical Protein/Baseplate Assembly Protein	61
Cluster-764	Hypothetical Protein	61
Cluster-104	Hypothetical Protein	62
Cluster-114	Head Maturation Protease	62
Cluster-209	Hypothetical Protein	62
Cluster-248	Hypothetical Protein/Putative Membrane Protein	62
Cluster-433	Hypothetical Protein	62
Cluster-436	Hypothetical Protein	62
Cluster-437	Hypothetical Protein	62
Cluster-438	Hypothetical Protein	62
Cluster-439	Hypothetical Protein	62
Cluster-441	Hypothetical Protein	62
Cluster-451	Hypothetical Protein	62
Cluster-453	Baseplate Assembly Protein	62
Cluster-488	DNA Primase/Helicase	62
Cluster-494	Exonuclease	62
Cluster-582	Baseplate Assembly Protein	62
Cluster-718	Major Capsid Protein	62
Cluster-405	Endolysin/Lysin	63
Cluster-434	Hypothetical Protein	63
Cluster-531	Ribonucleoside Triphosphate Reductase Beta Subunit	63
Cluster-673	rIIB Lysis Inhibitor	63
Cluster-617	DNA Ligase	64

Figure S1: Multiple sequence alignment of Lysin protein sequences. Lysins derived from the genomes of *Escherichia* Phage C600M2 (Protein ID: UCJ01465) and *Escherichia* Phage CL1 (Protein ID: UCJ01321) were aligned using MUSCLE Alignment tool[4] and visualized using ESPript[5].

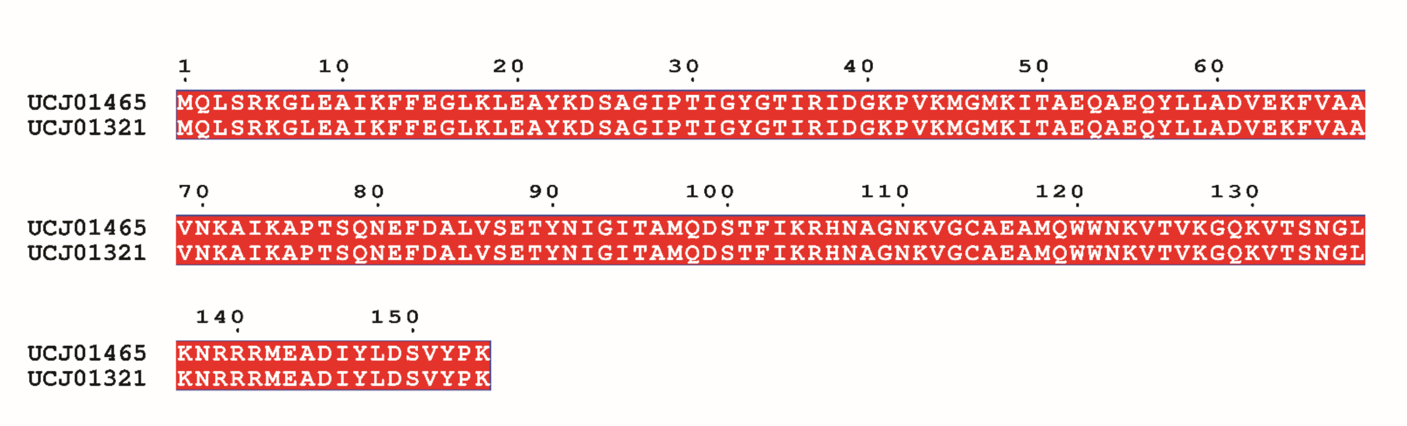

Figure S2: Ramachandran Plot. Generated using PROCHECK [6] for CL1-C600M2-Lysin protein model quality validation. Around 97.8% residues were present in the allowed region.

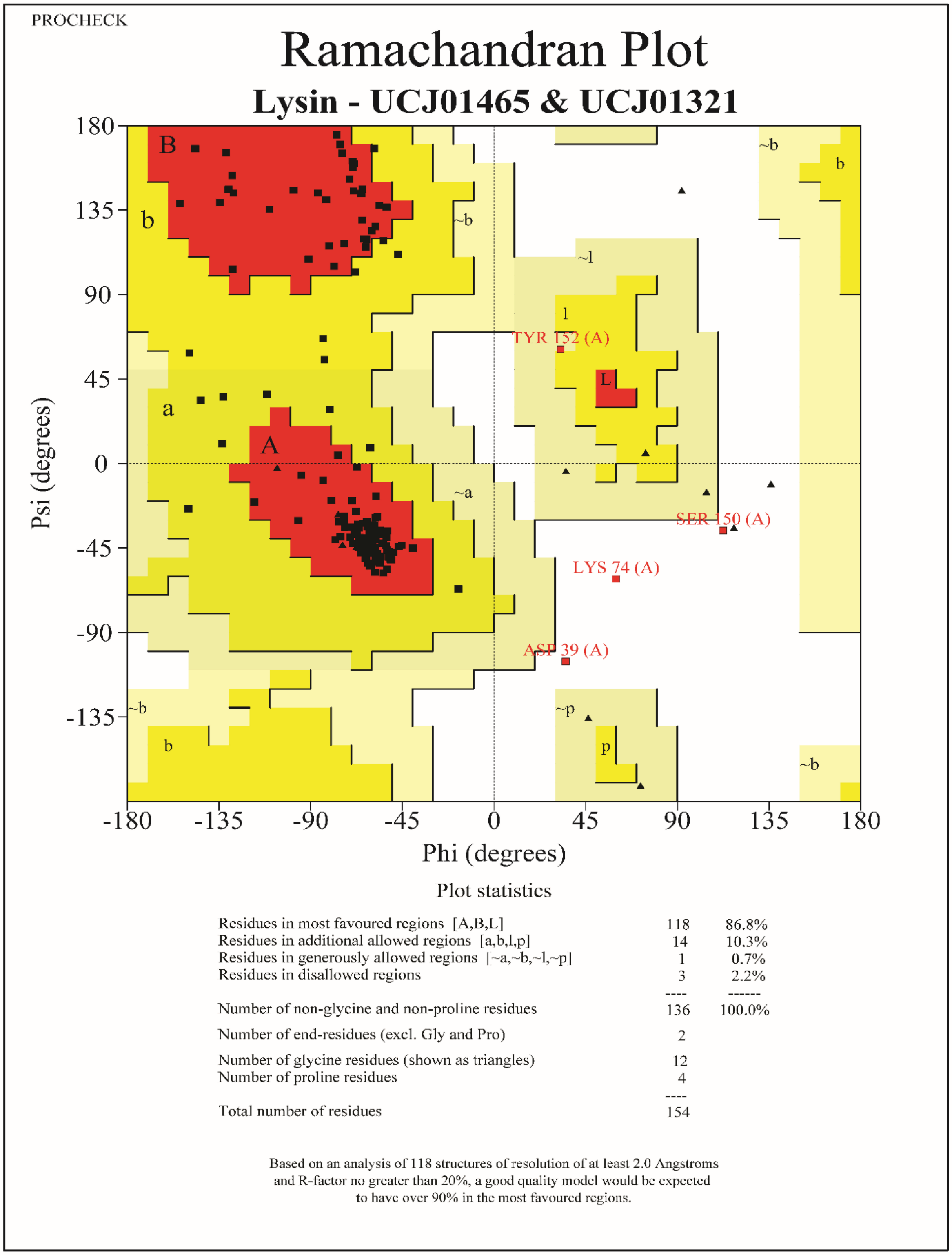

Figure S3: Multiple sequence alignment (MSA) of Protein-Blast hits: Generated in ESPript webserver. The residues of CL1-C600M2-Lysin interacting with WTA, TS and PG are highlighted as blue, green and pink dots respectively at the bottom of the MSA. Gly16, Leu17 and Arg18 of CL1-C600M2-Lysin interacts both with both TS and PG, hence the representing dots are overlapped and underlined with black line. Members of the Glu-Asp-Thr catalytic triad in the MSA are colored blue.

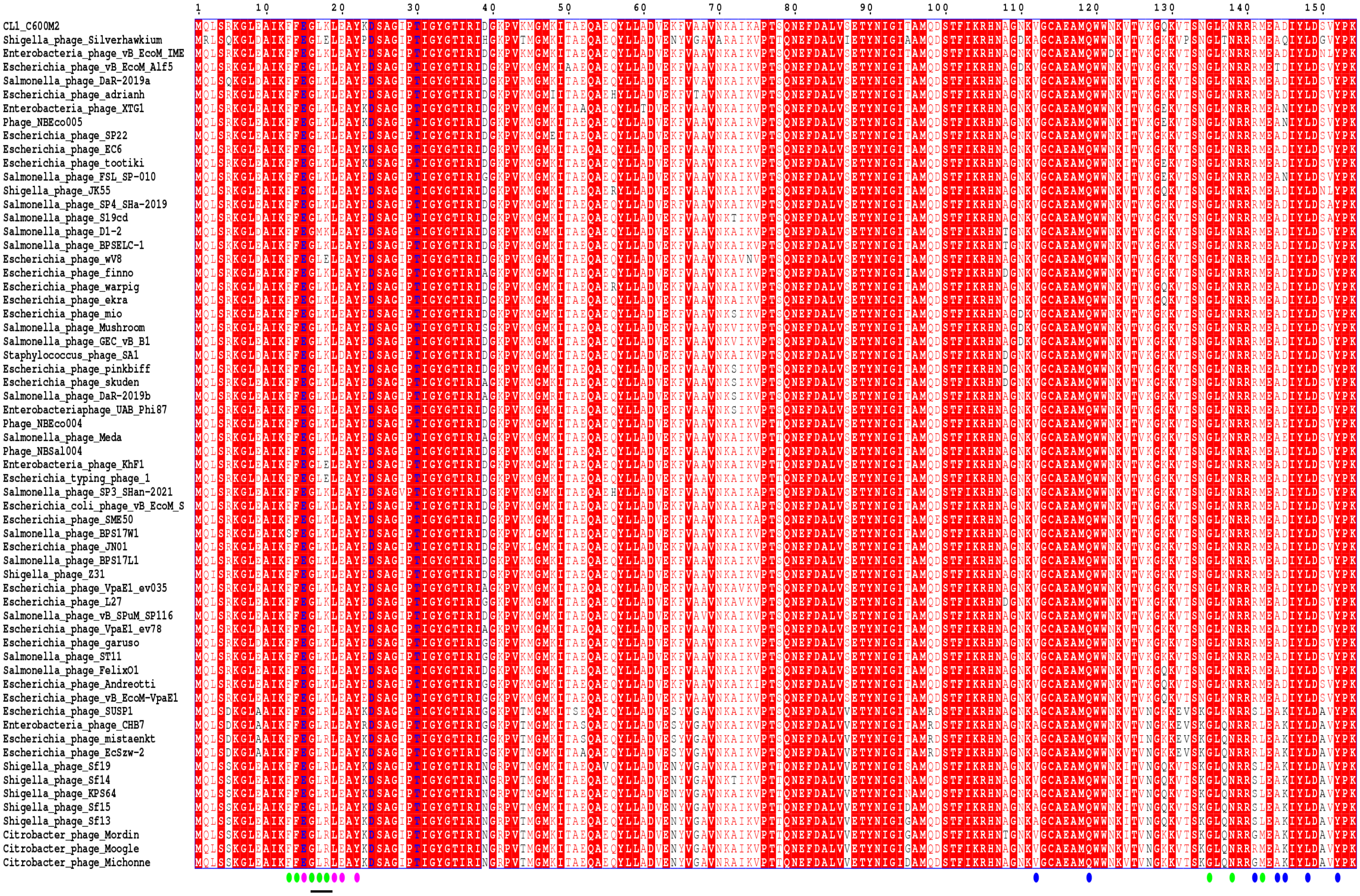

Figure S4: 2% Agarose gel electrophoresis to verify insertion of CL1-C600M2-Lysin. Cloned CL1-C600M2-Lysin within pRSET-emGFP was amplified by PCR using primers specific to the lysin sequence. Expected size of PCR product of about 500bp corresponding to the CL1-C600M2-Lysin was detected (Lane 7) estimated from the 1kb and 100bp ladder (New England Biolabs; N3232 & N3231; Lane 2 & 3).

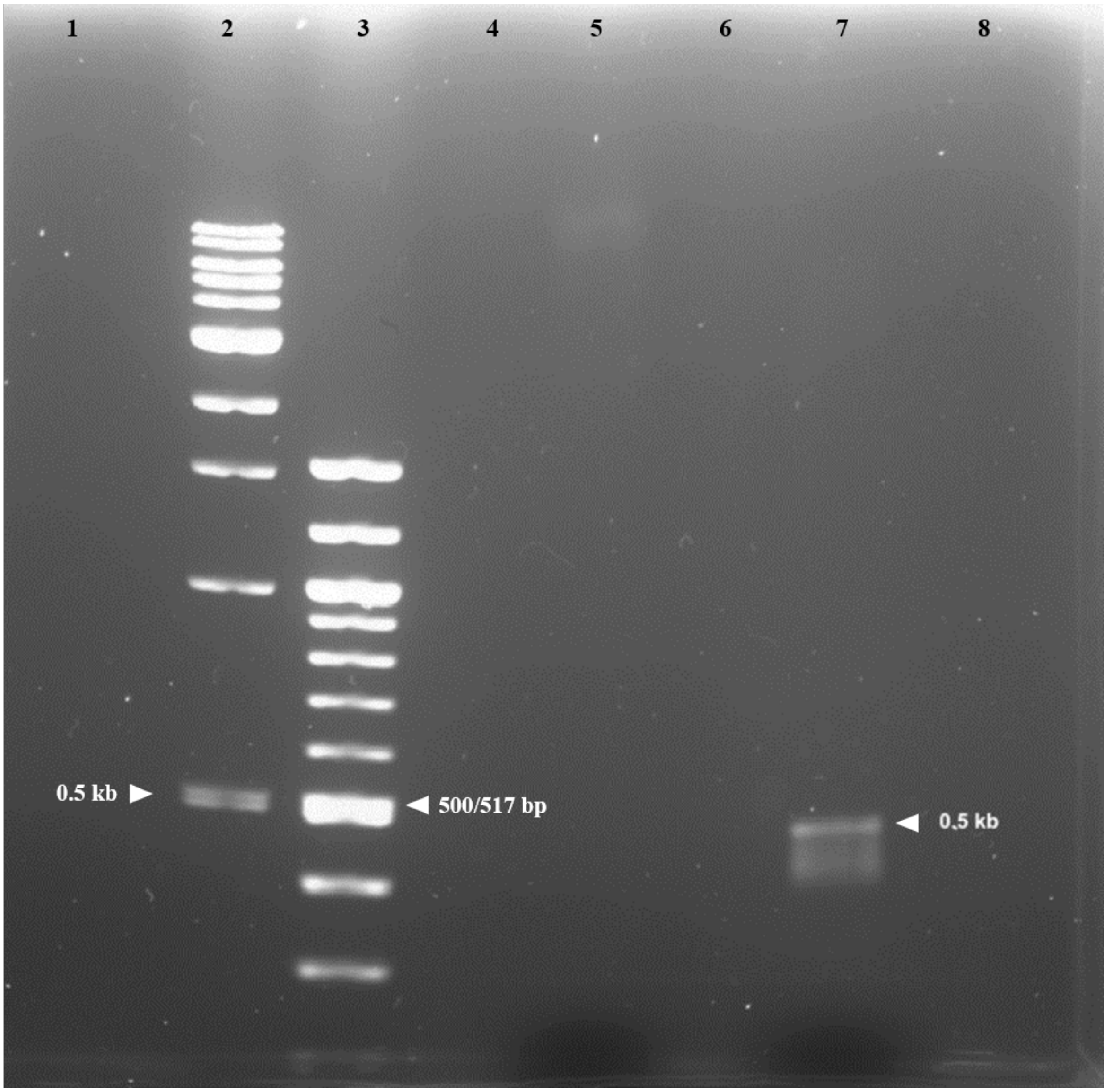

Figure S5: Confirmation by SDS-PAGE of expression and purity of CL1-C600M2-Lysin protein. The reported molecular weight of 21.27 kDa represent the *in silico* calculated molecular weight of the construct expressed. The 25 microliters of fractions obtained from each 1ml elution fraction was loaded (Lanes 2, 4, 6 – fractions 1 to 3 in order of elution) alongside 25ul of wash fraction (Lane 8) on 12.5% acrylamide gel which was stained with Coomassie blue. The size of the construct was estimated using the protein ladder in Lane 1 (New England Biolabs; P7712).

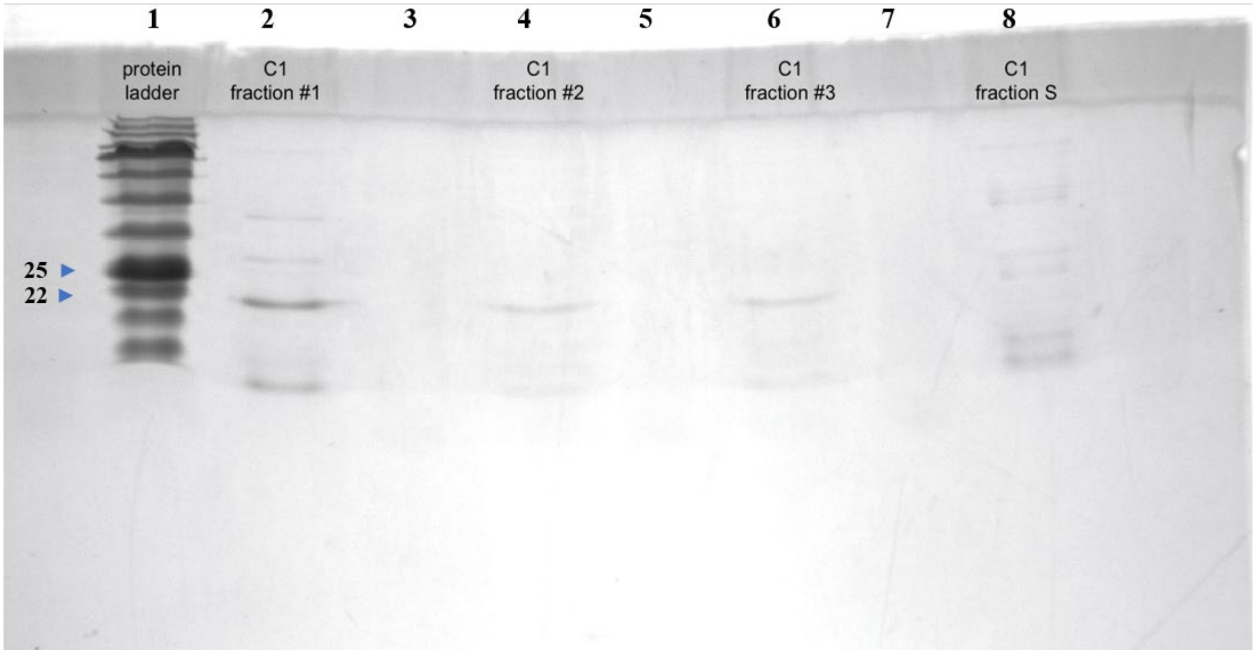

Supplementary Table 1: List of Bacteriophage lysins related to CL1-C600M2-Lysin. The details of GenBank accession IDs, %identity shared, Taxonomy of Source organism and the endemic environment are elaborated.

Supplementary Table 2: MMseqs2 [7] Output clusters from clustering analysis of proteome from Bacteriophages with related lysins. The details of clusters, GenBank accession IDs of the respective source organisms and protein sequences from their proteome are elaborated here.

References:

1. Lehar, S. M. *et al.* Novel antibody-antibiotic conjugate eliminates intracellular S. aureus. *Nature* (2015) doi:10.1038/nature16057.
2. Zhang, R. *et al.* Lysozyme's lectin-like characteristics facilitates its immune defense function. *Q. Rev. Biophys.* (2017) doi:10.1017/S0033583517000075.
3. Kuroki, R., Weaver, L. H. & Matthews, B. W. A covalent enzyme-substrate intermediate with saccharide distortion in a mutant T4 lysozyme. *Science (80-.).* **262**, 2030–2033 (1993).
4. Edgar, R. C. MUSCLE: a multiple sequence alignment method with reduced time and space complexity. *BMC Bioinformatics* **5**, 1–19 (2004).
5. Robert, X. & Gouet, P. Deciphering key features in protein structures with the new ENDscript server. *Nucleic Acids Res.* **42**, W320--W324 (2014).
6. Laskowski, R. A., MacArthur, M. W., Moss, D. S. & Thornton, J. M. PROCHECK: a program to check the stereochemical quality of protein structures. *J. Appl. Crystallogr.* **26**, 283–291 (1993).
7. Steinegger, M. & Söding, J. MMseqs2 enables sensitive protein sequence searching for the analysis of massive data sets. *Nat. Biotechnol.* **35**, 1026–1028 (2017).
